# Recovery from Emotion Recognition Impairment after Temporal Lobectomy

**DOI:** 10.3389/fneur.2014.00092

**Published:** 2014-06-06

**Authors:** Francesca Benuzzi, Giovanna Zamboni, Stefano Meletti, Marco Serafini, Fausta Lui, Patrizia Baraldi, Davide Duzzi, Guido Rubboli, Carlo Alberto Tassinari, Paolo Frigio Nichelli

**Affiliations:** ^1^Department of Biomedical, Metabolic and Neural Sciences, University of Modena and Reggio Emilia, Modena, Italy; ^2^OPTIMA Project, Nuffield Department of Clinical Medicine and FMRIB Centre, University of Oxford, Oxford, UK; ^3^Health Physics Department, AUSL Modena, Modena, Italy; ^4^IRCCS Institute of Neurological Sciences, Bellaria Hospital, Bologna, Italy; ^5^Danish Epilepsy Center, Epilepsihospitalet, Dianalund, Denmark

**Keywords:** mesial temporal lobe epilepsy, functional recovery, facial expression, emotion, functional magnetic resonance

## Abstract

Mesial temporal lobe epilepsy (MTLE) can be associated with emotion recognition impairment that can be particularly severe in patients with early onset seizures (1–3). Whereas, there is growing evidence that memory and language can improve in seizure-free patients after anterior temporal lobectomy (ATL) (4), the effects of surgery on emotional processing are still unknown. We used functional magnetic resonance imaging (fMRI) to investigate short-term reorganization of networks engaged in facial emotion recognition in MTLE patients. Behavioral and fMRI data were collected from six patients before and after ATL. During the fMRI scan, patients were asked to make a gender decision on fearful and neutral faces. Behavioral data demonstrated that two patients with early onset right MTLE were impaired in fear recognition while fMRI results showed they lacked specific activations for fearful faces. Post-ATL behavioral data showed improved emotion recognition ability, while fMRI demonstrated the recruitment of a functional network for fearful face processing. Our results suggest that ATL elicited brain plasticity mechanisms allowing behavioral and fMRI improvement in emotion recognition.

## Introduction

Mesial temporal lobe epilepsy (MTLE) is a drug-resistant epilepsy characterized by hippocampal sclerosis as well as by damage to the amygdala complex (5). The mesial temporal lobe structures are major components of a complex system involved in both memory and emotional functions. As a consequence, selective deficits in memory and emotional abilities have been described in MTLE patients. Surgery in MTLE has a notable advantage over medical treatment in terms of seizure control and subjectively reported quality of life (6). Although relatively little is known about the effect of surgery on cognitive functions, there is increasing evidence that functional compensation and reorganization can occur after epilepsy surgery (4).

The nature of the cognitive impairment in subjects with MTLE depends on the laterality of the damage: left-sided MTLE is generally characterized by material-specific verbal memory deficits, whereas right-sided MTLE is associated with deficits in spatial memory (7, 8), identification of famous faces (9), and recognition of emotion from facial expression (1, 3, 10).

In addition, cognitive impairment in subjects with MTLE depends on the age of onset of the seizures. It has been found that patients with seizures beginning before 5 years of age (early onset) have global (verbal and non-verbal) deficits, whereas patients with late onset seizures show lateralized memory deficits (11).

Despite the well-documented memory and emotional impairment in patients with MTLE, cognitive changes after temporal lobe surgery are not clear. It is known that left lobectomy affects verbal memory to varying degrees. Right lobectomy on the other hand, seems to carry a smaller risk for additional cognitive deficiencies or for exaggerating preoperative impairments (12, 13). However, long-term follow-up studies have demonstrated post-surgical improvements in memory and non-memory functions in temporal lobe epilepsy patients, indicating unexpectedly strong effects of plasticity and recovery (14, 15).

Several factors can affect the cognitive outcome of surgery. Early onset seizures seem to be a relevant issue in the case of language functions. Indeed, early onset left MTLE patients show significantly less post-operative decline in naming ability (16). Recently, a neuropsychological study demonstrated that early onset seizures are associated with better cognitive outcome compared to later onset in both right and left MTLE patients (17).

The effectiveness of epilepsy surgery in controlling the seizures is another important issue in determining the cognitive outcome. In a recent follow-up study, it has been demonstrated that seizure-free patients improve in non-memory functions within the first year after surgery. They also show a long-term recovery of the verbal memory decline that is common immediately after left-sided temporal surgery (14).

Functional imaging techniques have been successfully used to investigate mechanisms of brain plasticity, compensation, and reorganization, both in epilepsy and after epilepsy surgery, with existing reports focusing on memory and language.

The aim of the present study was to examine the cognitive and functional reorganization of emotion recognition processing in a group of patients with MTLE who underwent unilateral temporal lobectomy. Using functional magnetic resonance imaging (fMRI), we examined the impact of surgery on a specific neural network involved in the processing of facial expressions of fear. We previously evaluated 13 patients with MTLE, demonstrating that an early right mesial temporal lesion affected emotional processing (10). In the present study, we examined six of these patients, using the same fMRI protocol, both before and 6 months after unilateral temporal lobectomy. Among the six tested patients, two subjects with right MTLE and early seizure onset, who were impaired in emotion recognition prior to surgery, improved in a facial expression recognition test at 6 months after surgery. More interestingly, fMRI results showed the recruitment of a functional network for incidental processing of fearful facial expressions that was absent before surgery.

## Materials and Methods

### Subjects

Six right-handed (18) subjects (three males and three females) ranging in age from 20 to 27 years (mean age: 23.2 years), with no history of neurological or psychiatric illness, participated in the experiment as controls. They were re-tested after 6 months.

Six right-handed patients (one woman, five men; mean age: 36.8 years) with a history of drug-resistant MTLE, were also evaluated:

- Four right MTLE patients: two of them (T.D., V.M.) with MRI evidence of mesial temporal sclerosis (MTS), had a positive history for complex febrile convulsions during infancy and were considered to have suffered early damage to the temporal lobe (19). Patient G.C. had MTS but no history of complex febrile convulsions during infancy. Patient Z.A. had MRI evidence of lesion to the anteromesial temporal lobe other than MTS.
- Two left MTLE patients: patient B.D. with MRI evidence of MTS, and patient C.R. with lesions to the anteromesial temporal lobe other than MTS.

Each one of these patients underwent a highly uniform surgical procedure to control for medically intractable seizures, after a non-invasive presurgical evaluation performed at the Epilepsy Monitoring Unit of the Bellaria Hospital in Bologna, Italy. Pathology examination confirmed the diagnosis of MTS in patients T.D., V.M., B.D., and G.C., and of dysembrioplastic neuroepithelial tumor (DNET) in subject C.R. Post-surgical outcome was seizure-free (Engel class Ia) in all patients. The pharmacological treatment administered before lobectomy was continued for at least 1 year after the surgery.

All patients had been included in our previous study (10). A comprehensive battery of neuropsychological tests was administered to each patient. The Beck Depression Inventory was used to assess mood disturbances. Neuropsychological results and other clinical variables are shown in Table S1 in Supplementary Material. Functional and neuropsychological evaluations were performed both before and 6 months after unilateral temporal lobectomy.

Subjects’ consent was obtained according to the Declaration of Helsinki (20) and the study was approved by the Ethics Committee of the University of Modena and Reggio Emilia.

### Morphed facial expression test

Pictures were obtained using a computer graphics procedure that interpolates images along a continuum between two prototypes. We prepared 15 emotion continua to assess recognition of the 6 basic emotions (happiness, fear, surprise, anger, disgust, and sadness). Each continuum was obtained from the prototype faces of two emotions, morphed in the following proportions: 90–10, 70–30, and 50–50%. The continua were created using the faces of two individuals (JJ and SW, male and female, respectively) taken from the Ekman and Friesen (21) series. The resulting 135 morphed faces were used in a forced-labeling task. The pictures were presented one at a time on a computer screen in a random order. The subject’s task was to decide which emotion the morphed image most resembled: happiness, fear, surprise, anger, disgust, or sadness. The SuperLab software (Cedrus Corporation, San Pedro, CA, USA) was used for stimulus presentation and response collection.

Normative data were collected from 20 healthy volunteers (10 male, 10 female) ranging in age from 21 to 30 years.

The number of prototypical responses to 90–10% images in the Morphed Facial Expression Test was taken as an index of recognition of the target emotion, and was calculated for each of the six basic emotions. Then, the rate of intrusions (incorrect labeling of images, citing emotions other than the ones used for morphing) was calculated for the five continua containing fearful faces. To test whether an individual’s score differed significantly from the normative control sample, we used a modified independent samples *t*-test (22). The statistical threshold was adjusted using the Bonferroni correction and set at *p* = 0.01.

### fMRI experiment: Stimuli and experimental design

The task consisted in an implicit processing of emotional expressions.

Pictures of nine individuals (four males, five females) were used. Each individual showed fearful [mean percentage of emotion recognition was 87.9% (21)] or neutral expressions. Control stimuli were prepared applying an Adobe Photoshop mosaic filter of 512 pixels to the face pictures, thus obtaining grayscale images (masks), formed by 8 × 11 squares, no longer recognizable as faces. Since we have previously demonstrated (1, 3, 10) that right MTLE patients with early onset are impaired in explicit recognition of fearful expression, we needed a task our patients could perform (23). Therefore, in the two experimental conditions, i.e., the presentation of faces with fearful (F) and neutral (N) expressions, subjects were required to decide the face gender by pressing one of two buttons. In the control condition (C), they were asked to decide whether or not a white square was present in the center of the mask.

The experiment comprised six sessions of eight separate blocks. In each session, two blocks were presented for each experimental condition (F and N), in randomized order. A total of four control blocks alternated with experimental blocks. In each block, eight different stimuli were presented for 3.3 s. Before the beginning of each block, written instructions were presented to inform the subjects of the task they had to perform.

### Image acquisition and data analysis

Images were acquired using a GE Signa HHS77 system at 1.5 T. Echo-planar images were collected using a single shot, blipped, gradient echo-planar pulse imaging (EPI). To maximize field homogeneity, fine manual pre-scan and localized shimming was performed at the beginning of the first session. Each BOLD-echo-planar volume scan consisted of 16 transverse slices (in plane matrix 64 × 64; voxel size 3.75 mm × 3.75 mm × 5 mm; TE = 40 ms; TR: 3380 ms). Seventy-two volumes were collected in each scanning session and each subject underwent six sessions for a total of 432 volumes. A blocked design was used and nine volumes were acquired in each block. In addition, a high-resolution T1-weighted anatomical image of each subject was acquired to allow anatomical localization.

Image analysis was performed using SPM99 (Wellcome Department of Cognitive Neurosciences, London, UK).

All functional volumes for each subject were realigned to the first volume acquired, then smoothed with an 8-mm full width maximum isotropic Gaussian kernel to improve the signal-to-noise ratio.

The three conditions were modeled with two temporal basis functions convolved with the hemodynamic response function. Condition effects were estimated according to the general linear model and regionally specific effects were compared using linear contrasts comparing the neutral (N) and fearful (F) faces with the control condition (masks). To identify regions activated in both experimental conditions (F and N), a conjunction analysis was performed. The statistical threshold was set at *p* < 0.05 for the conjunction analysis and *p* < 0.001 (uncorrected) for the contrast “fearful vs. neutral expression.”

Patients were analyzed singly and activated regions were located by superimposing the SPM map on the individual T1 volume. The volumes were then aligned with the Talairach and Tournoux stereotaxic space (24) using the AFNI package (25).

## Results

### Emotion recognition before and after lobectomy

In the pre-surgical data, a significant difference was found between the two right TLE patients with early damage to the temporal lobe (T.D. and V.M.) and controls. The results showed a greater number of intrusions in the fear – sadness continuum (T.D.: 83.3% of intrusions; *t* = 4.24; *p* < 0.001; estimated percentage of normal population falling below individual’s score = 99.99%; lower 95% confidence limit = 99.88%; upper 95% confidence limit = 100%), in the fear – surprise continuum (T.D.: 33.3% of intrusions; *t* = 32.2; *p* < 0.001; estimated percentage of normal population falling below individual’s score = 100%; lower 95% confidence limit = 100%; upper 95% confidence limit = 100%), and in the fear–anger continuum (V.M.: 83.3% of intrusions; *t* = 4.94; *p* < 0.001; estimated percentage of normal population falling below individual’s score = 100%; lower 95% confidence limit = 99.97%; upper 95% confidence limit on the percentage = 100%).

**Figure 1:**
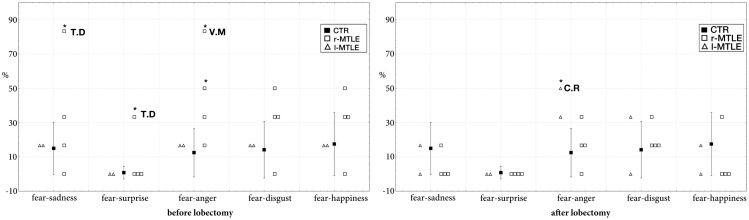
**Intrusion rate (percentage) in controls (mean ± SD) and in MTLE patients (individual scores) in the four continua containing fearful faces**. *Significantly different at *p* < 0.001.

Post-surgical data demonstrate that these right patients improved their performance in fearful recognition. The rate of intrusions of T.D. and V.M. after lobectomy did not significantly differ from controls (Figure 1). Defective performance was found only in one left MTLE: the rate of intrusions in the fear–anger continuum was significantly greater in patient C.R. (50% of intrusions; *t* = 2.65; *p* < 0.01 estimated percentage of normal population falling below individual’s score = 99.21%; lower 95% confidence limit = 95.95%; upper 95% confidence limit = 99.99%).

### Behavioral performance at the fMRI task

Accuracy and reaction times of the explicit task of gender recognition (for both fearful and neutral faces) and white square detection (for masks) are shown in Table S2 in Supplementary Material for both controls and patients.

No difference was found between controls and patients, and between the performances before and after surgery (Wilcox Match Paired Test, *p* < 0.016 Bonferroni correction).

### Neuroimaging results

#### Control subjects

##### Fearful and neutral expressions

In the fMRI data analysis, we first identified regions of activation common to fearful and neutral expressions (conjunction analysis: Table S3 in Supplementary Material). In particular, we examined regions previously found to be selective for processing visual information from faces (26), which also emerged in our previous study (10). The perception of faces (neutral and fearful expressions), compared to scrambled stimuli evoked activation in the lateral fusiform gyrus (face fusiform area or FFA), bilaterally, and mostly symmetrically. In addition, significant activations were found in other face responsive regions located in the inferior occipital gyrus and in the MT gyrus/ST sulcus. Similar activations were found both in the test and in the re-test experimental sessions.

##### Fearful vs. neutral expressions

The presentation of fearful compared to neutral expressions evoked activations in two extrastriate areas (Table S4 in Supplementary Material). Another significant region of this network was present in the orbitofrontal region. With respect to our previous study, we did not find activations in the lateral prefrontal cortex.

The specific activations found for fearful expressions changed between the first and the second session: re-test data did not show the extrastriate cortices activations found in the test session. We can speculate that this could reflect a long-term adaption of the emotional system to the incidental task, while the network for face processing did not show a similar effect.

#### MTLE patients

##### Fearful and neutral expressions

**Figure 2:**
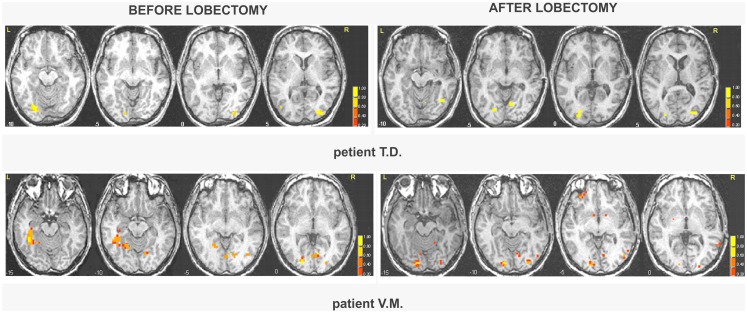
**Areas of significant activation in response to faces in right MTLE patients before and after lobectomy**. The numbers at the bottom of each sagittal slice represent *z*-axis levels according to Talairach coordinates. The statistical threshold is set at *p* < 0.001 (uncorrected). The activated clusters are superimposed on each single patient’s structural image.

All patients showed activations resembling the face-selective pattern found in controls (Tables 1 and 2; Tables S5 and S6 in Supplementary Material). The perception of faces compared to scrambled stimuli evoked activation in the lateral fusiform gyrus (FFA), bilaterally, and mostly symmetrically. In addition, significant activation was found in other face responsive regions located in the inferior occipital gyrus and in the MT gyrus/ST sulcus. Post-operative data showed activations of the same face-selective areas found before lobectomy. The specific pattern of activation for faces in patients with right MTLE associated with early mesial temporal lobe damage (T.D. and V.M.) are shown in Figure 2.

**Table 1:** **Main activated regions for faces before and after lobectomy in right MTLE patients**.

		G.C.		Z.A.	
		Before	After	Before	After
Right hemisphere	FFA	+	+	+	+
	Inferior occipital area	+	+	+	+
	MT gyrus/ST sulcus	+	+	+	+
Left hemisphere	FFA	+	+	+	+
	Inferior occipital areas	+	+	+	±
	MT gyrus/ST sulcus	+		+	+

		**T.D**.		**V.M**.	
		**Before**	**After**	**Before**	**After**

Right hemisphere	FFA	+	+		
	Inferior occipital area	+	+	+	+
	MT gyrus/ST sulcus			+	+
Left hemisphere	FFA	+		+	
	Inferior occipital areas	+	+	+	+
	MT gyrus/ST sulcus				

*Presence (+) of activation in each region of interest (fusiform face area, inferior occipital face responsive region, and MT gyrus/ST sulcus) for each patient. Details are presented in Table S5 in Supplementary Material*.

**Table 2:** **Main activated regions for faces before and after lobectomy in left MTLE patients**.

		B.D.		C.R.	
		Before	After	Before	After
Right hemisphere	FFA	+	+	+	+
	Inferior occipital area	+	+	+	+
	MT gyrus/ST sulcus	+	+		+
Left hemisphere	FFA	+	+	+	+
	Inferior occipital areas	+	+	+	+
	MT gyrus/ST sulcus		+		

*Presence (+) of activation in each region of interest (fusiform face area, inferior occipital face responsive region, and MT gyrus/ST sulcus) for each patient. Details are presented in Table S6 in Supplementary Material*.

##### Fearful vs. neutral expressions

The presentation of fearful compared to neutral expression evoked activations in temporo-occipital region, in the lateral prefrontal cortex and in the orbitofrontal region (Tables S7 and S8 in Supplementary Material). Similar activations were found in our previous fMRI study in controls and MTLE patients (10).

##### Right MTLE

**Figure 3:**
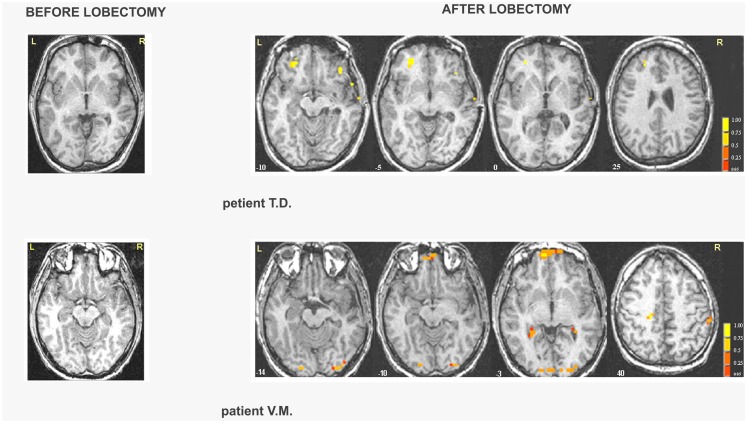
**Areas of significant activation in response to fearful expressions in right MTLE patients before and after lobectomy**. The numbers at the bottom of each sagittal slice represent *z*-axis levels according to Talairach coordinates. The statistical threshold is set at *p* < 0.001 (uncorrected) with a spatial extension of at least four contiguous voxels.

In patient G.C., activations were located in the occipital cortex before lobectomy and in the frontal cortex after lobectomy (Table S7 in Supplementary Material; Figure 3). In patient Z.A., a widespread network was found before surgery but only unilateral frontal and occipital clusters were found after lobectomy. Activation of distinct regions for processing fearful faces was absent in patients with right MTLE associated with early mesial temporal lobe damage (T.D. and V.M.) before lobectomy. These patients were also impaired in recognizing fear from facial expressions. Functional data showed that after lobectomy, both the occipital and the frontal areas were activated in patients T.D. and V.M while processing fearful faces (Table 3; Table S7 in Supplementary Material; Figure 3).

**Table 3:** **Main activated regions for fearful faces before and after lobectomy in right MTLE patients**.

		G.C.		Z.A.	
		Before	After	Before	After
Right hemisphere	Lateral prefrontal cortex			+	
	Orbitofrontal cortex		+	+	+
	Extrastriate cortices	+		+	
Left hemisphere	Lateral prefrontal cortex			+	
	Orbitofrontal cortex		+	+	
	Extrastriate cortices	+		+	+

		**T.D**.		**V.M**.	
		**Before**	**After**	**Before**	**After**

Right hemisphere	Lateral prefrontal cortex		+		
	Orbitofrontal cortex		+		
	Extrastriate cortices		+		+
Left hemisphere	Lateral prefrontal cortex				+
	Orbitofrontal cortex		+		+
	Extrastriate cortices		+		+

*Presence (+) of activation in each region of interest (lateral prefrontal cortex, orbitofrontal cortex, and extrastriate cortices) for each group of patients. Details are presented in Table S7 in Supplementary Material*.

##### Left MTLE

In patient B.D., there were activations bilaterally in some of these regions, whereas after lobectomy the whole set of regions showed activations, bilaterally (Table 4; Table S8 in Supplementary Material; Figure 3). In patient C.R., activations before surgery were located mostly in the left hemisphere, whereas after lobectomy both the frontal and the occipital cortex were recruited on the right side.

**Table 4:** **Main activated regions for fearful faces before and after lobectomy in left MTLE patients**.

		B.D.		C.R.	
		Before	After	Before	After
**LEFT MTLE PATIENTS**					
Right hemisphere	Lateral prefrontal cortex	+	+		+
	Orbitofrontal cortex		+	+	
	Extrastriate cortices		+		+
Left hemisphere	Lateral prefrontal cortex		+		
	Orbitofrontal cortex	+	+	+	
	Extrastriate cortices	+	+	+	

*Presence (+) of activation in each region of interest (lateral prefrontal cortex, orbitofrontal cortex, and extrastriate cortices) for each group of patients. Details are presented in Table S8 in Supplementary Material*.

## Discussion

This study concerns the functional bases of emotion recognition in a group of patients with MTLE, examined both before, and 6 months after temporal lobectomy. The present results show that the functional network for processing faces is not significantly affected either by epilepsy or temporal lobectomy. In fact, results of the conjunction analysis showed that MTLE patients activate a specific neural circuit for processing faces, similar to the one found in controls and in our previous study (10). Lobectomy, whether right or left, did not significantly affect this pattern. This is consistent with neuropsychological studies (4, 10), which found deficits in memory and language abilities, but did not find any impairment in face recognition in this group of patients.

Right MTLE patients have been previously found to be impaired in emotional processing. This deficit was present in implicit fear recognition (1, 27), correlated with the age of onset of epilepsy (3), and was associated with lack of a specific fMRI activation pattern for implicit processing of fearful facial expression (10).

In agreement with these findings, our pre-surgical data showed that only patients with early onset of seizures and presumably with damage to the right medial temporal lobe structures were impaired in the explicit recognition of emotional facial expression (Morphed Facial Expression Test). In functional MRI data, they did not present the typical pattern of activation for incidental processing of fearful faces (10), which includes occipito-temporal regions, the inferior frontal cortex (BA 44, BA 45, and BA 46) and the fusiform gyrus. These activations could reflect a neuromodulatory effect of the amygdala on extrastriate cortical regions (28–30). The absence of significant activations in patients with early medial right temporal damage (including the amygdala), supports the hypothesis that the integrity of the right amygdala is essential for the activation of these regions.

After lobectomy, in patients with right MTLE and early onset seizure (T.D. and V.M.) both the occipital and the frontal areas of the right hemisphere were activated in response to fearful faces. In particular, both T.D. and V.M. activated the left orbitofrontal cortex and extrastriate visual areas of both hemispheres. Results from the behavioral task showed that these two patients recovered the ability to recognize fear. These data suggest that successful temporal lobectomy can activate the process of functional compensation and plasticity in early onset MTLE. Since only decreased activations, in term of number and extension of clusters, could be found in the control group and in the other left and right MTLE patients, the recruitment of areas in right MTLE patients could not be attribute simply to a re-testing effect.

It is generally assumed that functional reorganization decreases with age, although the exact time window for efficient functional compensation of cognitive abilities is not known. Lesion studies focusing on language related areas concluded that the earlier a circumscribed lesion occurs, the better the outcome will be (4). However, this might not be true for epilepsy-related damage. Indeed epilepsy is a process, which can lead to a progressive deterioration with an additional indirect impairment of functional compensation in non-epileptic areas (11, 31). In particular, chronically discharging epileptic foci with brain damage occurring early in development can affect the brain’s potential for functional reorganization (32). Amygdalar–hippocampal dysfunction in MTLE is a progressive and dynamic pathology (33). Thus the impairment in emotion recognition found in right MTLE before surgery may be attributable both to the lesion *per se* and to the epileptic activity. Early onset seizure is associated with an increasing number of years of intractable seizure activity in the temporal lobe and with the propagation of the epileptic discharge into extratemporal regions. Ictal and interictal epileptogenic activity could modify neural networks from the temporo-mesial region, resulting in functional and behavioral disturbances (10).

After successful right MTLE surgery, the removal of the chronically discharging epileptic foci may activate reorganization processes, including the recruitment of a widespread functional network comprising the left orbitofrontal cortex and extrastriate visual areas of both hemispheres. There is evidence supporting the notion that the orbitofrontal cortex plays a critical role in emotional and social behavior (34–36), while the extrastriate visual regions are part of the neuromodulatory network supported by the amygdala in response to negative facial expressions (29). Thus, the activation of this network in right MTLE patients after lobectomy reflects the improvement in explicit recognition of fearful facial expressions.

PET studies consistently demonstrated hypometabolism in frontal regions in MTLE patients (37, 38). After successful surgery, patients show normalization of metabolism in the frontal cortex as well as in the thalamus (39). Following surgical removal of the medial temporal lobe, metabolic changes have been reported also in the contralateral mesial temporal lobe; they may reflect recovery of preoperative impaired contralateral temporal functions (40), in particular of memory and attentional abilities. Post-surgical improvement was found in seizure-free patients within the first year after surgery. In the same study, patients also showed a long-term recovery of the verbal memory decline that is common after left lobectomy (14). In addition, intracranial EEG recordings support the view that the orbitofrontal cortex is a preferential pathway for mesial temporal lobe seizure propagation (41). The activation of the orbitofrontal region we found could reflect this fronto-temporal interaction and the release of the functionality of this region after the resection of the epileptogenic zone.

Few studies have evaluated functional changes before and after temporal lobectomy, and they mainly focused on language functions. Preoperative functional imaging studies have shown ipsilateral and contralateral activations associated with language processing in both pediatric and adult epileptic patients (42–44). Post-operative plasticity of the language functional network involves either interhemispheric or intrahemispheric functional reorganization of language specific areas (45–47).

Summing up, behavioral studies demonstrated post-surgical cognitive improvement in MTLE patients who were seizure-free after surgery, confirming that plasticity and recovery occur even in the adult brain. So far, very few studies have used functional imaging before and after surgery, and they mainly focused on memory and language (47, 48).

In our study, we found that subjects with right MTLE and early seizure onset, who were impaired in emotion recognition, improved in a facial expression recognition test within the first 6 months after surgery. fMRI results showed a recruitment of a functional network comprising both frontal and extrastriate visual areas. Further evidences are needed, but from the present data it can be speculated that the removal of the chronically discharging epileptic foci might elicit reorganization processes necessary to the improvement of emotional recognition abilities.

## Conflict of Interest Statement

The authors declare that the research was conducted in the absence of any commercial or financial relationships that could be construed as a potential conflict of interest.

## Supplementary Material

The Supplementary Material for this article can be found online at http://www.frontiersin.org/Journal/10.3389/fneur.2014.00092/abstract
