# Supplementary material for: Recovery from Emotion Recognition Impairment after Temporal Lobectomy
Source: Front Neurol. 2014 Jun 6;5:92. doi: 10.3389/fneur.2014.00092 (PMC4047513; doi:10.3389/fneur.2014.00092)
Supplement: Supplementary file 1 [file DataSheet_1.ZIP › Table S1.DOCX]

***Supplementary Material***

**Recovery from emotion recognition impairment**

**after temporal lobectomy**

Francesca Benuzzi^1^*****, Giovanna Zamboni^2^, Stefano Meletti^1^, Marco Serafini^3^, Fausta Lui^1^, Patrizia Baraldi^1^, Davide Duzzi^1^, Guido Rubboli^4,5^, Carlo Alberto Tassinari^4^, Paolo Frigio Nichelli^1^

^1^ Department of Biomedical, Metabolic and Neural Sciences, University of Modena and Reggio Emilia, Modena, Italy

^2^OPTIMA Project, Nufﬁeld Department of Clinical Medicine and FMRIB Centre, University of Oxford, UK

^3^ Health Physics Dept., A.U. S. L. Modena, Modena, Italy

^4^ IRCCS Institute of Neurological Sciences, Bellaria Hospital, Bologna, Italy

^5^Danish Epilepsy Center, Epilepsihospitalet, Dianalund, Denmark.

*** Correspondence:** Dr. Francesca Benuzzi, Ph.D.

Department of Biomedical, Metabolic and Neural Sciences

University of Modena and Reggio Emilia

N.O.C.S.A.E. Hospital

Via Giardini 1355, Baggiovara

41126 Modena, Italy

phone : +39- 0593961679

fax: +39- 0593962409

e-mail: [francesca.benuzzi@unimore.it](mailto:francesca.benuzzi@unimore.it)

1. **Tables**

## Suplementary Tables

***Supplementary Table 1*** *Demographic and neuropsychological characteristics of MTLE patients*

**Right TLE patients**

|  | **G.C.** | | **Z.A.** | | **T.D.** | | **V.M** | |
| --- | --- | --- | --- | --- | --- | --- | --- | --- |
|  | **before** | **after** | **before** | **after** | **before** | **after** | **before** | **after** |
| **Sex** | M | | M | | F | | M | |
| **Age** | 37 | | 29 | | 36 | | 41 | |
| **Education (years)** | 13 | | 8 | | 13 | | 8 | |
|  |  |  |  |  |  |  |  |  |
| ***Digit Span** | 3.5 | 4.5 | 4.5 | 5.75 | 5.75 | 6.75 | 3.5 | 4.75 |
| ***Corsi Block Test** | 5.5 | 4.5 | 4.5 | 3.5 | 3.75 | 2.75 | 2.5 | 3.5 |
| ***Corsi Supraspan** | 17.14 | 5 | 4.5 | 5.52 | 2.28 | 7.68 | 3.23 | 0.75 |
| ***Babcock Story Recall Test** | 10.5 | 13.59 | 7 | 13.75 | 4.5 | 6.5 | 5.25 | 8.75 |
| ***Token Test** | 28 | 28 | 32 | 31.75 | 30.5 | 30 | 30.75 | 33.75 |
| **Judgment of Line Orientation** | 24 | 27 | 15 | 23 | 29 | 28 | 26 | 25 |
| ***Raven’s Colored Progressive Matrices** | 33 | 33.5 | 24.5 | 29 | 31.5 | 31 | 31 | 32 |
| **Beck Depression Inventory** | 14 | 5 | 3 | 1 | 1 | 0 | 21 | 0 |

**Left TLE patients**

|  | **B.D.** | | **C.R.** | |
| --- | --- | --- | --- | --- |
|  | **before** | **after** | **before** | **after** |
| **Sex** | M | | M | |
| **Age** | 41 | | 37 | |
| **Education (years)** | 17 | | 13 | |
|  |  |  |  |  |
| ***Digit Span** | 3.25 | 3.25 | 5.5 | 6.5 |
| ***Corsi Block Test** | 3.5 | 2.5 | 3.5 | .35 |
| ***Corsi Supraspan** | 0 | 3.3 | 9.31 | 12.09 |
| ***Babcock Story Recall Test** | 6.5 | 7.75 | 10 | 3.5 |
| ***Token Test** | 25.75 | 30.25 | 30.5 | 28.5 |
| **Judgment of Line Orientation** | 28 | 29 | 30 | 29 |
| ***Raven’s Colored Progressive Matrices** | 28.25 | 30.25 | 33.75 | 32 |
| **Beck Depression Inventory** | 13 | 13 | 21 | 20 |

* corrected for age, sex and education
