# Supplementary material for: Recovery from Emotion Recognition Impairment after Temporal Lobectomy
Source: Front Neurol. 2014 Jun 6;5:92. doi: 10.3389/fneur.2014.00092 (PMC4047513; doi:10.3389/fneur.2014.00092)
Supplement: Supplementary file 1 [file DataSheet_1.ZIP › Table S2.DOCX]

***Supplementary Material***

**Recovery from emotion recognition impairment**

**after temporal lobectomy**

Francesca Benuzzi^1^*****, Giovanna Zamboni^2^, Stefano Meletti^1^, Marco Serafini^3^, Fausta Lui^1^, Patrizia Baraldi^1^, Davide Duzzi^1^, Guido Rubboli^4,5^, Carlo Alberto Tassinari^4^, Paolo Frigio Nichelli^1^

^1^ Department of Biomedical, Metabolic and Neural Sciences, University of Modena and Reggio Emilia, Modena, Italy

^2^OPTIMA Project, Nufﬁeld Department of Clinical Medicine and FMRIB Centre, University of Oxford, UK

^3^ Health Physics Dept., A.U. S. L. Modena, Modena, Italy

^4^ IRCCS Institute of Neurological Sciences, Bellaria Hospital, Bologna, Italy

^5^Danish Epilepsy Center, Epilepsihospitalet, Dianalund, Denmark.

*** Correspondence:** Dr. Francesca Benuzzi, Ph.D.

Department of Biomedical, Metabolic and Neural Sciences

University of Modena and Reggio Emilia

N.O.C.S.A.E. Hospital

Via Giardini 1355, Baggiovara

41126 Modena, Italy

phone : +39- 0593961679

fax: +39- 0593962409

e-mail: [francesca.benuzzi@unimore.it](mailto:francesca.benuzzi@unimore.it)

|  |  | | **Before lobectomy** | | | | | | **After lobectomy** | | | | | |  |
| --- | --- | --- | --- | --- | --- | --- | --- | --- | --- | --- | --- | --- | --- | --- | --- |
|  |  | | *Fearful faces* | | *Neutral faces* | | *Masks* | | *Fearful faces* | | *Neutral faces* | | *Masks* | |  |
|  | **G.C.** | | 100%  0,899 | | 100%  0,878 | | 99,58%  0,800 | | 97,92%  0,961 | | 100%  0,965 | | 97,65%  0,899 | |  |
|  | **Z.A.** | | 100%  0,613 | | 100%  0,576 | | 98,89%  0,600 | | 100%  1,229 | | 96,25%  1,220 | | 100%  1,197 | |  |
|  | **T.D.** | | 97,92%  0,809 | | 100%  0,805 | | 96,45%  0,895 | | 100%  0,775 | | 98,96%  0,772 | | 99,57%  0,798 | |  |
|  | **V.M.** | | 100%  0,746 | | 100%  0,871 | | 97,50%  0,769 | | 100%  0,933 | | 98,94%  0,929 | | 95,34%  0,933 | |  |
|  |  | |  | |  | |  | |  | |  | |  | |  |
| **left MTLE** | **B.D.** | | 100%  0,741 | | 100%  0,811 | | 99,32  0,719 | | 96,88%  0,882 | | 100%  0,877 | | 97,33%  0,675 | |  |
|  | **C.R.** | | 100%  1,122 | | 98,44%  1,083 | | 94,78%  1,023 | | 100%  1,029 | | 100%  1,079 | | 100%  1,104 | |  |
|  |  | |  | |  | |  | |  | |  | |  | |  |
|  |  | | Test | | | | | | Retest | | | | | |  |
|  | | | *Fearful faces* | | *Neutral faces* | | *Masks* | | *Fearful faces* | | *Neutral faces* | | *Masks* | |  |
| **CTR** | | | 99,82%  0,693 | | 99,65%  0,663 | | 99,51%  0,636 | | 99,3%  0,675 | | 99,3%  0,676 | | 99,6%  0,635 | |  |
|  | |  | |  | |  | |  | |  | |  | |  | |

***Supplementary Table 2*** *Accuracy and response time in the gender discrimination task*
