# Supplementary material for: Recovery from Emotion Recognition Impairment after Temporal Lobectomy
Source: Front Neurol. 2014 Jun 6;5:92. doi: 10.3389/fneur.2014.00092 (PMC4047513; doi:10.3389/fneur.2014.00092)
Supplement: Supplementary file 1 [file DataSheet_1.ZIP › Table S3.DOCX]

***Supplementary Material***

**Recovery from emotion recognition impairment**

**after temporal lobectomy**

Francesca Benuzzi^1^*****, Giovanna Zamboni^2^, Stefano Meletti^1^, Marco Serafini^3^, Fausta Lui^1^, Patrizia Baraldi^1^, Davide Duzzi^1^, Guido Rubboli^4,5^, Carlo Alberto Tassinari^4^, Paolo Frigio Nichelli^1^

^1^ Department of Biomedical, Metabolic and Neural Sciences, University of Modena and Reggio Emilia, Modena, Italy

^2^OPTIMA Project, Nufﬁeld Department of Clinical Medicine and FMRIB Centre, University of Oxford, UK

^3^ Health Physics Dept., A.U. S. L. Modena, Modena, Italy

^4^ IRCCS Institute of Neurological Sciences, Bellaria Hospital, Bologna, Italy

^5^Danish Epilepsy Center, Epilepsihospitalet, Dianalund, Denmark.

*** Correspondence:** Dr. Francesca Benuzzi, Ph.D.

Department of Biomedical, Metabolic and Neural Sciences

University of Modena and Reggio Emilia

N.O.C.S.A.E. Hospital

Via Giardini 1355, Baggiovara

41126 Modena, Italy

phone : +39- 0593961679

fax: +39- 0593962409

e-mail: [francesca.benuzzi@unimore.it](mailto:francesca.benuzzi@unimore.it)

1. **Tables**

## Suplementary Tables

***Supplementary Table 3:*** *Main activated regions for faces in controls in the two experimental sessions*

Coordinates of maximum voxel in each region of interest (Fusiform Face Area, inferior occipital face responsive region and MT gyrus/ ST sulcus) for the two experimental sessions: Test and Re-test (6 months after). For each activate region the Talairach coordinates (x, y, z), size of the overall activation (mm^3^) and T-value are given.

|  |  | **Test** | **Re-test** |
| --- | --- | --- | --- |
| right  hemisphere | **FFA** | 40, -51, -18  16576* (>8) | 36, -52, -21  16128* (>8) |
|  | **inferior occipital area** | 36, -82, -9  16576* (>8) | 32, -82, -13  16128* (>8) |
|  | **MT gyrus /ST sulcus** | 51, -58, 0  16576* (5.31) | 44, 77, -11  16128* (6.46) |
|  |  |  |  |
| left  hemisphere | **FFA** | -44, -55, -17  20096* (>8) | -44, -55, -17  19648*(>8) |
|  | **inferior occipital areas** | -40, -78, -10  20096* (>8) | -28, -82, -13  19648*(>8) |
|  | **MT gyrus /ST sulcus** | -55, -66, 7  1216 (6.72) | -55, -70, 3  19648*(6.14) |
