# Supplementary material for: Recovery from Emotion Recognition Impairment after Temporal Lobectomy
Source: Front Neurol. 2014 Jun 6;5:92. doi: 10.3389/fneur.2014.00092 (PMC4047513; doi:10.3389/fneur.2014.00092)
Supplement: Supplementary file 1 [file DataSheet_1.ZIP › Table S4.DOCX]

***Supplementary Material***

**Recovery from emotion recognition impairment**

**after temporal lobectomy**

Francesca Benuzzi^1^*****, Giovanna Zamboni^2^, Stefano Meletti^1^, Marco Serafini^3^, Fausta Lui^1^, Patrizia Baraldi^1^, Davide Duzzi^1^, Guido Rubboli^4,5^, Carlo Alberto Tassinari^4^, Paolo Frigio Nichelli^1^

^1^ Department of Biomedical, Metabolic and Neural Sciences, University of Modena and Reggio Emilia, Modena, Italy

^2^OPTIMA Project, Nufﬁeld Department of Clinical Medicine and FMRIB Centre, University of Oxford, UK

^3^ Health Physics Dept., A.U. S. L. Modena, Modena, Italy

^4^ IRCCS Institute of Neurological Sciences, Bellaria Hospital, Bologna, Italy

^5^Danish Epilepsy Center, Epilepsihospitalet, Dianalund, Denmark.

*** Correspondence:** Dr. Francesca Benuzzi, Ph.D.

Department of Biomedical, Metabolic and Neural Sciences

University of Modena and Reggio Emilia

N.O.C.S.A.E. Hospital

Via Giardini 1355, Baggiovara

41126 Modena, Italy

phone : +39- 0593961679

fax: +39- 0593962409

e-mail: [francesca.benuzzi@unimore.it](mailto:francesca.benuzzi@unimore.it)

1. **Tables**

## Suplementary Tables

***Supplementary Table 4:*** *Main activated regions for fearful faces in controls in the two experimental sessions*

Coordinates of maximum voxel in each region of interest (orbitofrontal cortex, and extrastriate cortices) for the two experimental sessions: Test and Re-test (6 months after). For each activate region the Talairach coordinates (x, y, z), size of the overall activation (mm^3^) and Z score are given.

|  |  | **Test** | **Re-test** |
| --- | --- | --- | --- |
| right  hemisphere | **lateral prefrontal cortex** |  |  |
|  | **orbitofrontal cortex** | 40 55 5  1152 (4.04) |  |
|  | **extrastriate cortices** | 24 -67 -10  320 (3.37) |  |
|  |  |  |  |
| left  hemisphere | **lateral prefrontal cortex** |  |  |
|  | **orbitofrontal cortex** | -36 59 8  1472 (4.58) | -20 42 -12  512 (3.98) |
|  | **extrastriate cortices** | -32 -63 -10  320 (3.26) |  |
