# Supplementary material for: Recovery from Emotion Recognition Impairment after Temporal Lobectomy
Source: Front Neurol. 2014 Jun 6;5:92. doi: 10.3389/fneur.2014.00092 (PMC4047513; doi:10.3389/fneur.2014.00092)
Supplement: Supplementary file 1 [file DataSheet_1.ZIP › Table S5.DOCX]

***Supplementary Material***

**Recovery from emotion recognition impairment**

**after temporal lobectomy**

Francesca Benuzzi^1^*****, Giovanna Zamboni^2^, Stefano Meletti^1^, Marco Serafini^3^, Fausta Lui^1^, Patrizia Baraldi^1^, Davide Duzzi^1^, Guido Rubboli^4,5^, Carlo Alberto Tassinari^4^, Paolo Frigio Nichelli^1^

^1^ Department of Biomedical, Metabolic and Neural Sciences, University of Modena and Reggio Emilia, Modena, Italy

^2^OPTIMA Project, Nufﬁeld Department of Clinical Medicine and FMRIB Centre, University of Oxford, UK

^3^ Health Physics Dept., A.U. S. L. Modena, Modena, Italy

^4^ IRCCS Institute of Neurological Sciences, Bellaria Hospital, Bologna, Italy

^5^Danish Epilepsy Center, Epilepsihospitalet, Dianalund, Denmark.

*** Correspondence:** Dr. Francesca Benuzzi, Ph.D.

Department of Biomedical, Metabolic and Neural Sciences

University of Modena and Reggio Emilia

N.O.C.S.A.E. Hospital

Via Giardini 1355, Baggiovara

41126 Modena, Italy

phone : +39- 0593961679

fax: +39- 0593962409

e-mail: [francesca.benuzzi@unimore.it](mailto:francesca.benuzzi@unimore.it)

1. **Tables**

## Suplementary Tables

***Supplementary Table 5:*** *Main activated regions for faces before and after lobectomy in right MTLE patients*

Coordinates of maximum voxel in each region of interest (Fusiform Face Area, inferior occipital face responsive region and MT gyrus/ ST sulcus) for each patient. For each activate region the Talairach coordinates (x, y, z), size of the overall activation (mm^3^) and Z score are given. Superscript numbers indicated that the same cluster include different region; * p< 0.001 uncorrected.

|  |  | **G.C.** | | **Z.A.** | |
| --- | --- | --- | --- | --- | --- |
|  |  | **before** | **after** | **before** | **after** |
| right  hemisphere | **FFA** | 37 -42 -17  504 (5.69) | 31 -43 -19  289 (5.20) | 30 -45 -10  1155 (>8) | 26 -65 -11  3538 (>8) |
|  | **inferior occipital area** | 45 -66 -10  2455 (>8) | 36 -62 -1  5054 ( >8) | 12 -79 3  144 (5.18) | 11 -90 11  288,8 (5.47) |
|  | **MT gyrus /ST sulcus** | 50 -55 20  939 (7.44) | 38 -55 23  289 (3.55)* | 42 -54 15  722 (4.32)* | 47 -55 5  361 (5.53) |
|  |  |  |  |  |  |
| left  hemisphere | **FFA** | -33 -38 -17  1155 (>8) | -37 -45 -24  1444 (>8) | -35 -44 -9  72 (4.52) | -37 -43 -8  433 (7.60) |
|  | **inferior occipital areas** | -42 -71 -7  1516 (>8) | -20 -70 -21  1372 (>8) | -43 -61 7  578 (4.10)* | -25 -74 -5  505 (>8) |
|  | **MT gyrus /ST sulcus** | -37 -51 -21  289 (3.84)* |  | -53 -33 7  217 (3.89)* | -46 -58 -9  72 (3.12)* |

|  |  | **T.D.** | | **V.M** | |
| --- | --- | --- | --- | --- | --- |
|  |  | **before** | **after** | **before** | **after** |
| right  hemisphere | **FFA** | 46 -42 -14  217 (3.56)* | 40 -58 -11  144 (5.05) |  |  |
|  | **inferior occipital area** | 26 -84 8  939 (6.74) | 32 -81 -3  217 (5.08) | 24 -84 2  144 (4.66) | 32 -85 -7  433 (4.25)* |
|  | **MT gyrus /ST sulcus** |  |  | 53 -55 -3  505 (4.28)* | 55 -53 4  433 (3.68)* |
|  |  |  |  |  |  |
| left  hemisphere | **FFA** | -31 -40 -19  433 (6.53) |  | -24 -50 -9  2671 (5.93) |  |
|  | **inferior occipital areas** | -25 -73 -13  939 (6.19) | -25 -86 -1  578 (5.78) | -20 -80 -2  433 (7.25) | -15 -86 -13  361 (6.05) |
|  | **MT gyrus /ST sulcus** |  |  |  |  |
