# Supplementary material for: Recovery from Emotion Recognition Impairment after Temporal Lobectomy
Source: Front Neurol. 2014 Jun 6;5:92. doi: 10.3389/fneur.2014.00092 (PMC4047513; doi:10.3389/fneur.2014.00092)
Supplement: Supplementary file 1 [file DataSheet_1.ZIP › Table S6.DOCX]

***Supplementary Material***

**Recovery from emotion recognition impairment**

**after temporal lobectomy**

Francesca Benuzzi^1^*****, Giovanna Zamboni^2^, Stefano Meletti^1^, Marco Serafini^3^, Fausta Lui^1^, Patrizia Baraldi^1^, Davide Duzzi^1^, Guido Rubboli^4,5^, Carlo Alberto Tassinari^4^, Paolo Frigio Nichelli^1^

^1^ Department of Biomedical, Metabolic and Neural Sciences, University of Modena and Reggio Emilia, Modena, Italy

^2^OPTIMA Project, Nufﬁeld Department of Clinical Medicine and FMRIB Centre, University of Oxford, UK

^3^ Health Physics Dept., A.U. S. L. Modena, Modena, Italy

^4^ IRCCS Institute of Neurological Sciences, Bellaria Hospital, Bologna, Italy

^5^Danish Epilepsy Center, Epilepsihospitalet, Dianalund, Denmark.

*** Correspondence:** Dr. Francesca Benuzzi, Ph.D.

Department of Biomedical, Metabolic and Neural Sciences

University of Modena and Reggio Emilia

N.O.C.S.A.E. Hospital

Via Giardini 1355, Baggiovara

41126 Modena, Italy

phone : +39- 0593961679

fax: +39- 0593962409

e-mail: [francesca.benuzzi@unimore.it](mailto:francesca.benuzzi@unimore.it)

1. **Tables**

## Suplementary Tables

***Supplementary Table 6:*** *Main activated regions for faces before and after lobectomy in left MTLE patients*

Coordinates of maximum voxel in each region of interest (Fusiform Face Area, inferior occipital face responsive region and MT gyrus/ ST sulcus) for each patient. For each activate region the Talairach coordinates (x, y, z), size of the overall activation (mm^3^) and Z score are given. Superscript numbers indicated that the same cluster include different region; * p< 0.001 uncorrected.

|  |  | **B.D.** | | **C.R.** | |
| --- | --- | --- | --- | --- | --- |
|  |  | **before** | **after** | **before** | **after** |
| right  hemisphere | **FFA** | 34 -29 -15  4404^2^(>8) | 38 -30 -15  5559^3^ (>8) | 37 -41 -9  144 (6.35) | 38 -42 -6  578 (6.98) |
|  | **inferior occipital area** | 33 -65 -6  4404^2^ (6.05) | 42 -68 -4  5559^3^ (6.80) | 36 -69 -9  1805 (>8) | 29 -89 1  578^4^ (6.41) |
|  | **MT gyrus /ST sulcus** | 51 -67 11  505 (5.92) | 47 -63 16  72 (4.60) |  | 42 -59 -3  578^4^ (6.41) |
|  |  |  |  |  |  |
| left  hemisphere | **FFA** | -35 -33 -21  72 (4.65) | -28 -55 -9  1949^4^ (>8) | -35 -56 -15  289 (6.53) | -34 -57 -14  886 (7.17) |
|  | **inferior occipital areas** | -34 -61 -10  1227 (6.85) | -44 -66 -2  1949^4^ (5.02) | -23 -88 -9  650*(6.92) | -17 -87 -1  432 (6.01) |
|  | **MT gyrus /ST sulcus** |  | -56 -33 0  505 (5.75) |  |  |
