# Supplementary material for: Recovery from Emotion Recognition Impairment after Temporal Lobectomy
Source: Front Neurol. 2014 Jun 6;5:92. doi: 10.3389/fneur.2014.00092 (PMC4047513; doi:10.3389/fneur.2014.00092)
Supplement: Supplementary file 1 [file DataSheet_1.ZIP › Table S8.DOCX]

***Supplementary Material***

**Recovery from emotion recognition impairment**

**after temporal lobectomy**

Francesca Benuzzi^1^*****, Giovanna Zamboni^2^, Stefano Meletti^1^, Marco Serafini^3^, Fausta Lui^1^, Patrizia Baraldi^1^, Davide Duzzi^1^, Guido Rubboli^4,5^, Carlo Alberto Tassinari^4^, Paolo Frigio Nichelli^1^

^1^ Department of Biomedical, Metabolic and Neural Sciences, University of Modena and Reggio Emilia, Modena, Italy

^2^OPTIMA Project, Nufﬁeld Department of Clinical Medicine and FMRIB Centre, University of Oxford, UK

^3^ Health Physics Dept., A.U. S. L. Modena, Modena, Italy

^4^ IRCCS Institute of Neurological Sciences, Bellaria Hospital, Bologna, Italy

^5^Danish Epilepsy Center, Epilepsihospitalet, Dianalund, Denmark.

*** Correspondence:** Dr. Francesca Benuzzi, Ph.D.

Department of Biomedical, Metabolic and Neural Sciences

University of Modena and Reggio Emilia

N.O.C.S.A.E. Hospital

Via Giardini 1355, Baggiovara

41126 Modena, Italy

phone : +39- 0593961679

fax: +39- 0593962409

e-mail: [francesca.benuzzi@unimore.it](mailto:francesca.benuzzi@unimore.it)

1. **Tables**

## Suplementary Tables

***Supplementary Table 8*** *Main activated regions for fearful faces before and after lobectomy in left MTLE patients*

Coordinates of maximum voxel in each region of interest (lateral prefrontal cortex, orbitofrontal cortex, and extrastriate cortices) for each group of patients. For each activate region the Talairach coordinates (x, y, z), size of the overall activation (mm^3^) and Z score are give

**Left MTLE patients**

|  |  | **B.D.** | | **C.R.** | |
| --- | --- | --- | --- | --- | --- |
|  |  | **before** | **after** | **before** | **after** |
| right  hemisphere | **lateral pref. cortex** | 44 46 -5  144 (3.63) | 42 26 -11  1011 (4.09) |  | 36 28 -6  72 (3.16) |
|  | **orbitofrontal cortex** |  | 20 59 0  289 (5.30) | 8 55 -8  722 (5.55) |  |
|  | **extrastriate cortices** |  | 26 -61 -7  1011 (3.96) |  | 36 -72 6  72 (3.22) |
|  |  |  |  |  |  |
| left  hemisphere | **lateral pref. cortex** |  | -32 27 -19  866 (4.36) |  |  |
|  | **orbitofrontal cortex** | -13 47 -4  289 (5.00) | -13 -64 -1  504 (4.25) | -17 66 17  361 (3.73) |  |
|  | **extrastriate cortices** | -28 -28 18 | -46 -51 -18  505 (5.75) | -38 -68 -17 |  |
